# Evolutionary history of versatile-lipases from Agaricales through reconstruction of ancestral structures

**DOI:** 10.1186/s12864-016-3419-2

**Published:** 2017-01-03

**Authors:** Jorge Barriuso, María Jesús Martínez

**Affiliations:** Centro de Investigaciones Biológicas, Consejo Superior de Investigaciones Científicas, Department of Environmental Biology, Ramiro de Maeztu 9, Madrid, E-28040 Spain

**Keywords:** Enzyme ancestors, *Candida rugosa*-like lipases, Genome sequencing

## Abstract

**Background:**

Fungal “Versatile carboxylic ester hydrolases” are enzymes with great biotechnological interest. Here we carried out a bioinformatic screening to find these proteins in genomes from Agaricales, by means of searching for conserved motifs, sequence and phylogenetic analysis, and three-dimensional modeling. Moreover, we reconstructed the molecular evolution of these enzymes along the time by inferring and analyzing the sequence of ancestral intermediate forms.

**Results:**

The properties of the ancestral candidates are discussed on the basis of their three-dimensional structural models, the hydrophobicity of the lid, and the substrate binding intramolecular tunnel, revealing all of them featured properties of these enzymes. The evolutionary history of the putative lipases revealed an increase on the length and hydrophobicity of the lid region, as well as in the size of the substrate binding pocket, during evolution time. These facts suggest the enzymes’ specialization towards certain substrates and their subsequent loss of promiscuity.

**Conclusions:**

These results bring to light the presence of different pools of lipases in fungi with different habitats and life styles. Despite the consistency of the data gathered from reconstruction of ancestral sequences, the heterologous expression of some of these candidates would be essential to corroborate enzymes’ activities.

**Electronic supplementary material:**

The online version of this article (doi:10.1186/s12864-016-3419-2) contains supplementary material, which is available to authorized users.

## Background

Triacylglycerol lipases (EC 3.1.1.3) and sterol esterases (EC 3.1.1.13) are defined as enzymes that hydrolyze acylglycerols and sterol esters as their natural substrates, respectively [1]. They are able not only to catalyze hydrolysis reactions under aqueous conditions, but also to carry out synthesis reactions, such as esterification and transesterification, in the presence of organic solvents [2]. All of them display the α/β-hydrolase fold, with their catalytic machinery formed by a catalytic triad (serine, histidine and glutamic acid) and the oxyanion hole [3]. These enzymes are present from microbes to plants and animals, and are widely applicable in industrial processes, such as biofuels, oleochemical, food, detergents, cosmetics, pharmaceutical, textile and paper industry, due to their versatility and stability to harsh conditions [4].

Among filamentous fungi and yeasts, some extracellular proteins show wide substrate specificity and combine the properties from lipases and sterol esterases, being active towards acylglycerols and sterol esters [2, 5]. These enzymes belong to the *Candida rugosa*-like lipase family (abH03.01), and their reclassification as “Versatile lipases” has been recently proposed [6]. Structurally, they present a hydrophobic cavity covered by a mobile, amphipathic α-helix, named “lid” or “flap”, fixed by a disulfide bond. The difference in activity and substrate affinity towards triglycerides and sterol esters among the proteins of this family is explained by small changes in the hydrophobicity of these regions [7, 8]. Most of the fungi producing these versatile lipases are saprophytic ascomycetes, isolated from forest, agricultural, or composting soils [9]. The only lipase from this family reported in basidiomycetes belongs to a white-rot fungus from the order Agaricales, *Pleurotus sapidus*. This enzyme acts on alcohol esters, cinnamyl esters and xhantophyl esters [10].

Considering the potential of these enzymes in different biotechnological applications there is an increasing interest in their study and evolution. In this sense, the development of massive DNA sequencing and new bioinformatics tools, have allowed the mining of fungal genomes and metagenomes, especially those from public repositories, such as that from the Joint Genome Institute (JGI) from the US Department of Energy (DOE) (http://www.jgi.doe.gov/), which contains more than 563 accessible genomes from different fungi with potential biotechnological interest [8, 11].

Agaricales is the largest clade of mushroom-forming fungi [12]. This order comprises ectomycorrhizal species and wood saprophytes, such as white rot fungi, which are the only organisms capable of degrading lignin efficiently [13] leaving accessible wood carbohydrates, a major pool of organic carbon in the planet [14]. In addition, Agaricales are one of the most ancient fungal orders, with the last common ancestor of the *Agaricomycetidae* clade probably dating between 125 and 150 million years ago [15].

On the other hand, exploring the evolutionary history and resurrecting intermediate ancestral forms of enzymes can help to explain the mechanistic basis of enzymes function, to disclose new functionalities, and even to evolve artificially these ancestral proteins in laboratory conditions [16, 17]. Evolutionary studies have been used to reconstruct a growing number of ancestral proteins, including hormone receptors [18, 19], visual pigments [20, 21], carbohydrate binding proteins [22], and elongation factors [23, 24]. In contrast, few ancestral enzymes have been reconstructed [25–31].

Since the number of molecular fossils is very scarce, ancestral sequences are calculated using novel computational methods [16]. Phylogenetic approaches are the most frequently applied for this purpose and, at present, the maximum likelihood method is one of the most widely used [32–34]. Different software applications have been developed to conduct this kind of analysis, as FastML [35], ANSESCON [36], or GASP [37], but the most extensively reported in the literature is PAML [38].

In the present work we carried out a bioinformatics screening of public fungal genomes to explore the presence of genes encoding putative “Versatile lipases” from the order Agaricales. We reconstructed the molecular evolution of these enzymes and inferred the sequence of their ancestral intermediate forms. The potential properties of the candidates are discussed on the basis of their three-dimensional (3D) model structure, the presence and hydrophobicity of the lid, and the substrate binding tunnel. To our knowledge this is the first report on the reconstruction of sequences from ancestral lipases.

## Methods

### Genomes mining

Genes encoding putative versatile-lipases from the *C. rugosa*-like family were searched in public genomes from the Joint Genome Institute MycoCosm database (http://www.jgi.doe.gov/). Automatically predicted proteins from each of the available Agaricales genomes containing the terms “esterase” or “lipase” were downloaded using the Advanced Search option at the JGI web-site. On the other hand, a homology search was carried out for each of the same genomes using BLASTp against all the filtered model proteins (*e*-value of 10^−5^), using as query the model lipase 3 (Lip3) from *C. rugosa* (P32947), and the lipase from *Pleurotus sapidus* (CAH17527), the only *C. rugosa*-like lipase from basidiomycetes.

The pool of redundant sequences obtained from each individual genome was subjected to a phylogenetic analysis. An un-rooted tree was created for each pool of sequences using MUSCLE for multiple sequence alignment, and the Maximum-Likelihood method (MEGA 5.1). A set of reference sequences from different lipase families (*C. rugosa*-like family, *Yarrowia* family, brefeldin family, cellular organelles, pancreas and bacteria) was included. Sequences grouping with the versatile-lipases from the *C. rugosa*-like family were checked for the presence of the lid region and conserved motifs. These sequences were also compared in “The Lipase Engineering Data Project” (LED) database to belong to the family ab3.01 [5]. Putative signal peptides in each sequence were predicted using the SignalP 4.0 server.

### Sequence analysis

Three-dimensional models of the sequences of the selected putative enzymes were generated using the programs implemented by the automated protein homology-modeling server SWISS-MODEL (http://swissmodel.expasy.org/). The models were comprehensively analyzed using PyMol 1.1 (http://pymol.org/) to check for the presence of a lid, and the existence and orientation of the catalytic triad. Putative intramolecular tunnels were modeled using Caver analyst 1.0 [39] taking the catalytic serine in each candidate as starting point.

The candidates from genomes that fulfilled the sequence and structural features of the *C. rugosa*-like lipase family were pooled together. These candidates had predicted signal peptide, possessed a lid region, showed at least 30% sequence identity with the model lipase Lip3 from *C. rugosa*, and the automatically selected template in the 3D modeling program corresponded to the structure of an enzyme from this family.

A phylogenetic analysis of all selected putative lipases was carried out. Multiple sequence alignment was performed using MUSCLE (MEGA 5.1) and trimmed manually to eliminate the signal peptide and long gaps using BioEdit 7.1.11. The most suitable evolution model for the set of sequences was checked using ProtTest 2.4 server [40], and a Maximum-Likelihood tree was built using the WAG (+F) evolution model with 100 bootstrap repetitions (MEGA 5.1). The tree was rooted taking as outgroup the most divergent sequence (SCCO2696509).

### Ancestral reconstruction

To infer the sequence of the ancestral nodes generated in the previous tree, PAML4.8 software [38] was used with the following parameters: WAG substitution matrix, Empirical + F model, no gaps, amino acids sequence type and verbose detailed.

Ancestral nodes 128 and 129, and nodes from 155 to 162 were selected for further analysis. Ancestral sequences were aligned and checked manually. The lid region, which is hypervariable and polymorphic, varied from 23 to 52 residues in the sequences obtained from the genomes, while in all ancestral nodes the reconstructed sequences from the lid were composed of 52 residues due to the inference method. To adjust the lid to the most probable length in each of the ancestors, the probability for the presence of each of the amino acids in this region was examined. Residues with probabilities below a selected threshold in the matrix generated by PAML4.8 were deleted; specifically six different thresholds were assayed (65, 70, 75, 80, 85 and 90%).

The sequences inferred from the ancestral nodes were manually curated and subjected to three-dimensional modeling, and intramolecular tunnels prediction as described above. Furthermore, number of hydrophobic residues in the lid region was determined.

## Results

Predicted proteins from the genomes of 39 Agaricales available at JGI (November 2015; Additional file 1) were screened using the terms “esterase” or “lipase”, obtaining a total of 6772 and 2631 candidates, respectively. Furthermore, Blast analysis of the same genomes using *C. rugosa* Lip3 and *P. sapidus* lipase as a query produced 1055 and 1031 matches, respectively. Putative lipases obtained for each genome were subjected to further sequence and phylogenetic analysis; those belonging to the versatile-lipases from the *C. rugose*-like family were selected for the construction of homology 3D models.

Two hundred and thirty four putative lipases matched the selected criteria (prediction of signal peptide, sequence length and sequence identity higher than 30% to *C. rugosa* Lip3) (Additional file 1). The software for 3D structural modeling of these proteins automatically selected as templates the structures of *C. rugosa* Lip1, 2 or 3 (PDBs: 1cle, 1clr, 1gz7, 1llf, 1llp, 1thg, 1trh), or the *Ophiostoma piceae* lipase/sterol-esterase (PDBs: 4be4, 4be9, 4upd). All models presented the typical α/β-hydrolase fold, and the catalytic triad and oxyanionic hole were well orientated in the space.

The average number of lipases in the different genomes was analyzed. Ectomycorrhizal fungi and plant parasites would present the lowest number of total lipases (4.20 ± 1.05), while a significantly higher quantity (7.18 ± 0.64; *P* = 0.039) was found in species associated to wood habitats and litter-associated fungi had an intermediate number of these enzymes (4.83 ± 0.38).

**Fig. 1:**
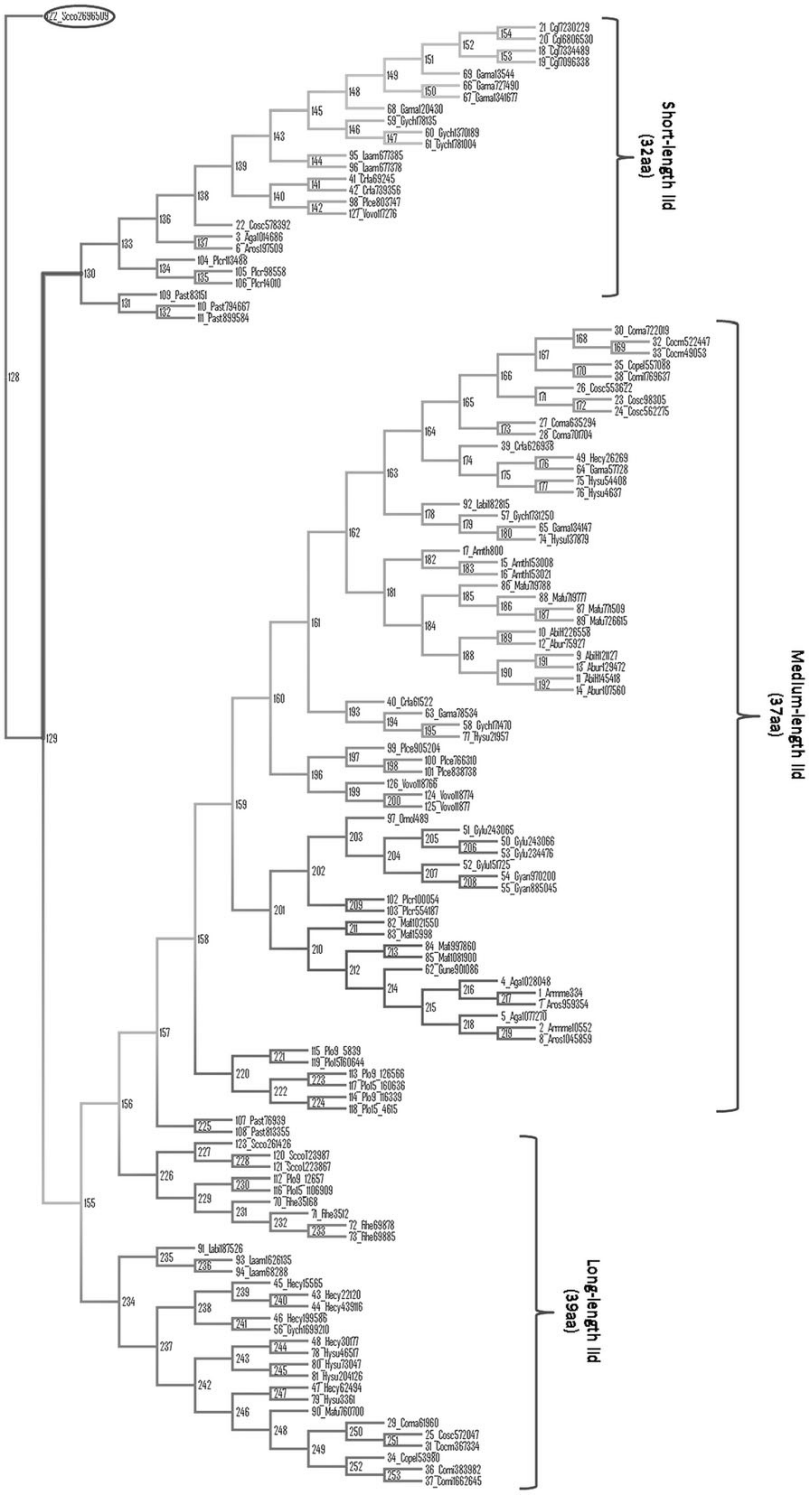
Phylogenetic tree of putative protein sequences of fungal versatile lipases. Phylogenetic tree of 127 putative versatile-lipases from genomes from fungi of the order Agaricales, and representative sequences of the *C. rugosa*-like family, *Yarrowia* family, *Brefeldin* family, cellular organelles, pancreatic sterol esterases, and bacterial esterases. This tree is rooted using sequence SCCO2696509 as outgroup. The numbers associated with the nodes are extant proteins used for ancestral reconstruction

Among the putative lipases, 107 presented less than 20 amino acids in the lid region and hence there was not an α-helix forming the flap. These were discarded to continue the study since all the active lipases described from the *C. rugosa*-like family have a lid (Additional file 1). The 127 remaining sequences from putative lipases were selected for further analysis (Additional file 2). These candidates were subjected to phylogenetic analysis, and ancestral sequences reconstruction was performed in all nodes of the tree (Fig. 1). In most of the cases, the candidates grouped independently of their fungal family or habitat, however, there was a clear clustering according to the number of residues in the lid region. The outgroup presented the lowest number of amino acids (23), while the most ancestral branch was formed by 26 putative proteins with a short lid, ranging from 24 to 38 residues (average length 32 amino acids). In the next branch on the evolutionary tree (Fig. 1) there was a very heterogeneous group of enzymes with lids ranging from 25 to 52 residues (average 39 amino acids). In addition, the most evolved groups were mainly formed by lipases with a lid of 37 amino acids (ranging from 36 to 38 amino acids).

Ancestral nodes 128 and 129, and those from 155 to 162 were selected as key intermediates in the evolution as origin of new groups (Fig. 1). The sequences of these ancestors were carefully studied, especially the lid region that presented strong polymorphisms. The most ancestral nodes presented the shorter lids when deleting residues with probability levels below 65, 70, 75, 80, 85 or 90%. Additional file 3 shows the sequences of nodes with residues in the lid region with a probability higher than 85%. The lid of the most ancient ancestor (128) was short, only 12 amino acids, however, the lid was longer in the following ancestors (129 and 155–162), containing sequences of 21, 26, 33, 35, 35, 36, 36, 35, and 35 residues, respectively. Moreover, when the number of hydrophobic amino acids in the lid region was analyzed, an increased percentage of these residues during evolution from ancestor 128 to 162 was observed: 41.6, 52.4, 50.0, 57.6, 57.1, 60.0, 61.1, 63.8, 62.8, and 62.8%, respectively.

The typical conserved motifs of the *C. rugosa*-like lipase family corresponding to the oxyanionic hole and the catalytic serine (GGGF–GESAG), were both present in all ancestral nodes, except for node 128 that contains the sequences GGGL–GQSAG.

**Fig. 2:**
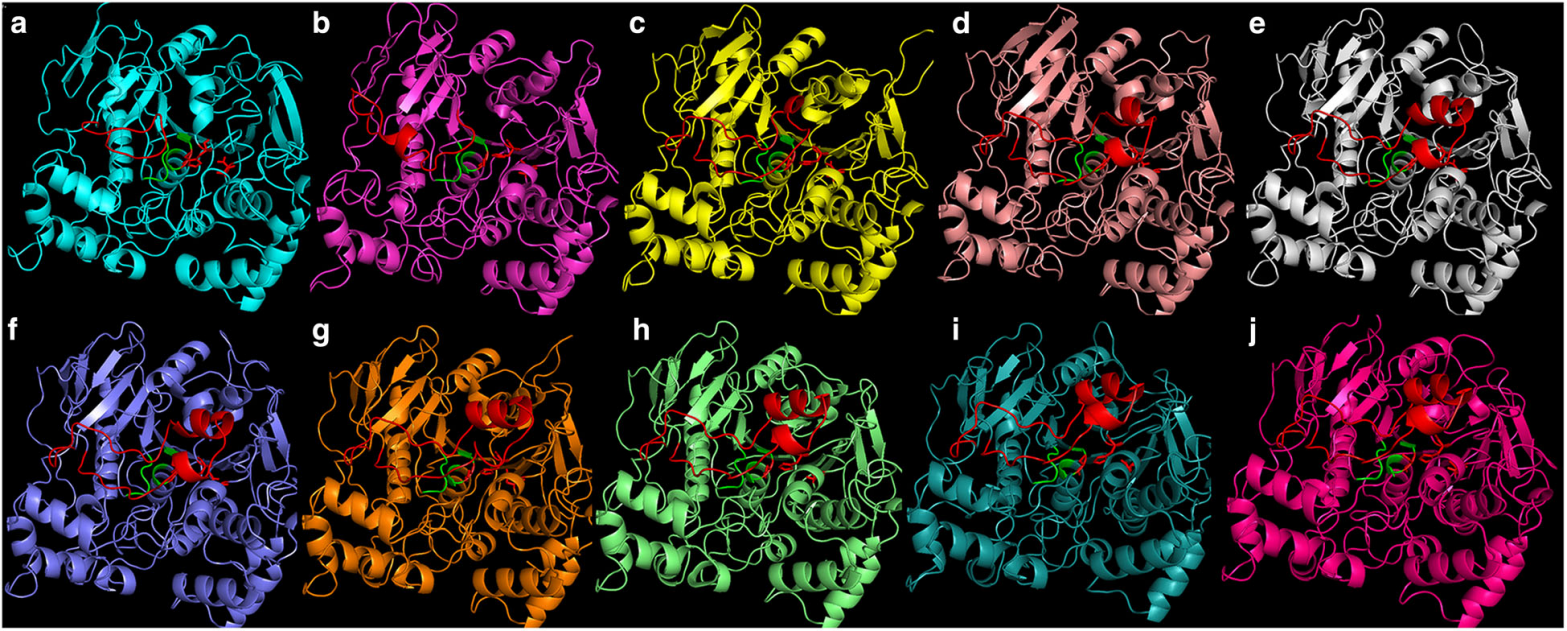
Tridimensional models of the putative ancestral versatile lipases. **a** Node 128, **b** Node 129, **c** Node 155, **d** Node 156, **e** Node 157, **f** Node 158, **g** Node 159, **h** Node 160, **i** Node 161 and **j** Node 162. The lid region and the three catalytic residues are indicated in *red*

The sequences from the ten ancestral nodes selected were subjected to 3D homology modeling (Fig. 2). All of them used Lip2 from *C. rugosa* as template (PDB 1gz7) except node 128 that used *O. piceae* lipase/sterol-esterase (PDB 4be4), with a Qmean value ranging from −6.20 to −9.86. All models had the typical α/β-hydrolase fold and the catalytic triad and oxyanionic hole correctly orientated in the space (Fig. 2). However, there were differences at the lid region; the older ancestors presented a loop instead of an α-helix, while more modern ancestors displayed the typical flap (Fig. 2).

**Fig. 3:**
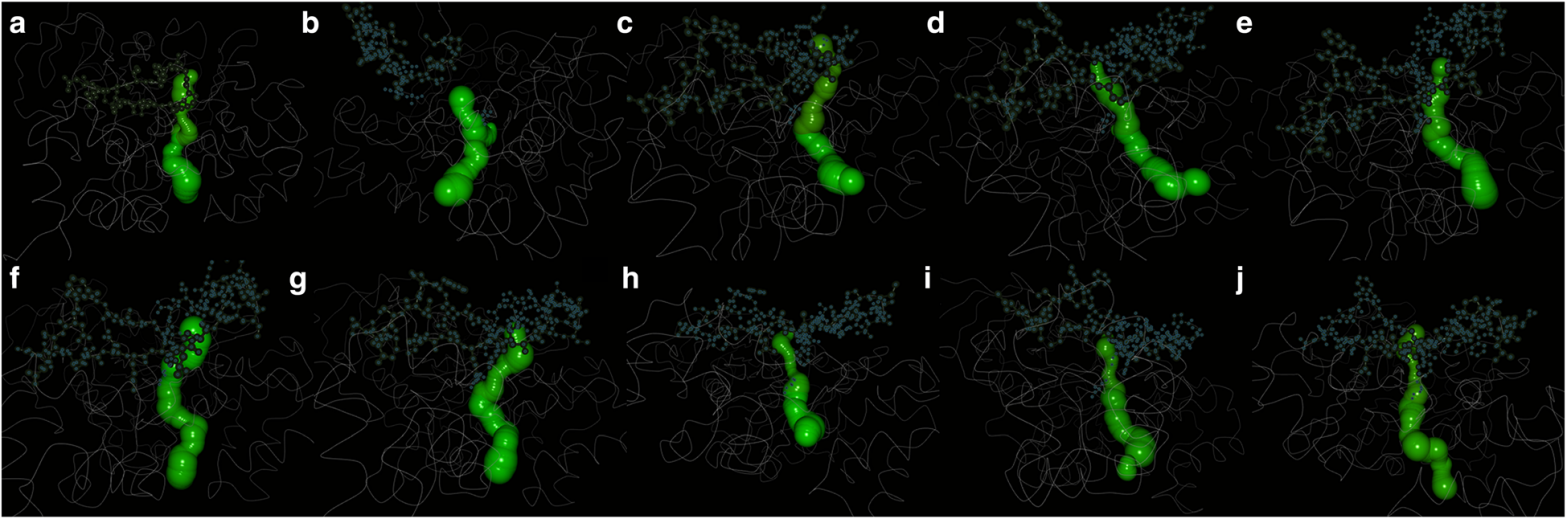
Models of the intramolecular tunnels of the putative ancestral versatile lipases. **a** Node 128, **b** Node 129, **c** Node 155, **d** Node 156, **e** Node 157, **f** Node 158, **g** Node 159, **h** Node 160, **i** Node 161 and **j** Node 162. Internal tunnels in each structure were modeled using Caver analyst 1.0

Finally, the prediction of intramolecular tunnels that could be responsible for the different catalytic properties reported in the *C. rugosa*-like proteins [8] was studied in detail in the 10 selected ancestors. The models depicted in Fig. 3 show that tunnels with a reduced size are expected in the more ancient enzymes, while longer tunnels were predicted for more modern proteins. Likewise, Table 1 shows that the length of the intramolecular tunnel, measured from the catalytic serine to the end of the tunnel, increases proportionally to the evolution time.

**Table 1:** Statistics of the intramolecular tunnels of the putative ancestral versatile lipases

Node	Lenght (Å)	Max. radius (Å)	Min. radius (Å)
128	17,8	2,88	1,55
129	18,5	3,2	2
155	17,8	2,7	1,85
156	21	2,4	1,75
157	21,5	3,2	2,1
158	23,5	2,7	1,95
159	19	3	2,1
160	32,5	2,8	1
161	37	2,8	1
162	29,8	2,65	1,5

## Discussion

The outcomes from the present work reveal the presence of a great number of putative proteins belonging to the *C. rugosa*-like family in Agaricales, an extensive group of fungi where only one lipase with activity on alcohol esters, cinnamyl esters and xanthophyll esters has been characterized [10]. The use of the well know versatile lipase from the ascomycete *C. rugosa*, with wide activity on triglycerides and sterol esters, or the lipase from the basidiomycete *P. sapidus* in the Blast analysis of the genomes, rendered a similar number of matches despite their low sequence identity (41%). After screening the candidates, the number of total lipases in each individual genome seems to be correlated to the habitat of the fungus, rather than with the family to which they belong. Wood decay fungi presented the highest number of putative versatile lipases. These microorganisms are essential to initiate lignocellulose degradation and complete the carbon cycle [41]. In particular, primary colonizers may need a battery of lipases with different affinities, as these substrates have different composition in lipids and extractives [42]. The presence of versatile lipases in litter-degrading fungi was lower, probably because soil litter has previously been colonized and partially degraded by other microorganisms [43].

On the other hand, ectomycorrhizal fungi and plant pathogens may have specialized during evolution for colonization of a specific host, with a defined cuticle composition, thus needing a smaller set of genes encoding lipases. This has already been observed in other groups of enzymes such as plant cell wall-degrading hydrolases [14].

Variations in the number of lipases in fungi with the same life-style may be related to their adaptations to more than one specific habitat. This is the case of *Hebeloma cylindrosporum* that is both a wood colonizer and a mycorrhizal fungus [44]. Furthermore, these intrinsic variations may also be due to different origins of evolution, *i.e.* ectomycorrhizal Agaricales may have had up to 11 events of specialization [45].

On the other hand, although all the known active lipases from the *C. rugosa*-like family have an α-helix in the lid region, the abundance of sequences (107) in the genomes screened with the absence of this structure, and its potential relationship with fungal habitat, may indicate some specific biological function for these putative proteins. Further studies on these enzymes are necessary to determine this hypothesis, which is a plausible one, as other lipase families include enzymes without lid with high activity on typical lipase substrates [4, 5, 46].

Phylogenetic analysis of the selected lipases showed that they clustered according to the length of their lid rather than to the genome or family where they belonged (Fig. 1). The group of lipases presenting a heterogeneous lid length (between 25 and 52 amino acids) could be evolutionary intermediates, a mixture of short- and long-lid enzymes, that further stabilized this sequence to 37 residues, as seen in the following branches of the tree. Furthermore, the longer lids (48–52 amino acids) may correspond to candidates with wrong introns prediction in the automatic annotation pipeline at the JGI.

Reconstruction of ancestral sequences of proteins has been used as a strategy to understand molecular evolution, and to test hypotheses about protein function [16]. However, protocols for ancestral sequence reconstruction require a precise model of evolutionary processes, being the accuracy of the reconstructed ancestral sequences critical for such studies [47]. In this work we reconstructed the internal nodes of the tree, inferring the sequences of the ancestral putative lipases that gave rise to the different evolutionary branches. Ten ancestral sequences were studied attending to their relevance generating new groups. The tridimensional models of the ancestral proteins rendered a typical α/β-hydrolase fold with the catalytic triad and the oxyanionic hole correctly orientated in the space, indicating the possible functionality of these enzymes (Fig. 2). The oldest protein, in node 128, is the origin of the sequence selected as outgroup, in accordance with its shorter flap (12 amino acids). In this sense, it is worthy to mention that the 107 lipases with a lid shorter than 20 amino acids grouped as a new branch from node 128 when included in the phylogenetic analysis (data not shown). The next ancestors (129, 155 and 156) had longer lids (21, 26 and 33 amino acids, respectively). Node 129 is the precursor of the group of lipases with short lids (average 32 amino acids) while nodes155 and 156 are the origin of the rest of the groups in the tree (Fig. 1). The number of residues in the lid of proteins in nodes 157 to 162 stabilizes between 35 and 36, being the origin of all “modern” lipases with 37 residues in the flap region. The gradual expansion in length of this sequence seems to lead to the formation of the α-helix in the lid across evolution from node 128 to 162 (Fig. 2).

In the same way, the hydrophobicity of the lid seems to increase during evolution, ranging from 41% in node 128 to 63% in the last three nodes (160, 161 and 162). These data suggest that hydrophobic insertions were selected and finally stabilized. In this sense, it is known that the lid participates in substrate recognition and that the number of hydrophobic amino acids in this region is related to enzyme-substrate specificity [7, 8]. Bulky substrates interact with the lid while the acyl-chain of the substrate is being processed in the hydrophobic pocket. Therefore, the flap’s length may be crucial for substrate recognition and specificity. This theory is in good agreement with others postulating the promiscuity of ancestral enzymes, *i.e.* processing several different substrates during the very first steps of their evolution [48, 49].

Comparison of the structures of different lipases from the *C. rugosa-*like family indicates the importance of both the shape and amino acid composition of the internal tunnel, and their relationship with the kinetic properties of these proteins [3, 7, 8]. The analysis of putative intramolecular tunnels in the ancestral lipases showed that its size increased along evolution (Fig. 3 and Table 1). The most ancestral lipases seem to have a smaller substrate-binding pocket, which is getting bigger in more evolved proteins. Particularly, the length of the predicted tunnels increases from node 128 to node 162 with only two exceptions (nodes 159 and 162). The minimum radius of the tunnels seems to increase from node 128 to 159, but decrease dramatically in the last three nodes. This could be explained by reasoning that in some enzymes of the family, the tunnel extension is long enough to communicate the end of the substrate-binding pocket with the outside of the protein, creating a narrow exit tunnel to release the reaction products [6, 8]. The evolutionary advantage of having a longer tunnel, as those found in some of the current proteins, could be related to a better accommodation of the acyl moiety of the substrate, increasing the specificity of the enzyme as reported for *C. rugosa* lipase isoenzymes and the lipase/esterase from *O. piceae* [2, 46].

## Conclusions

The search of “Versatile lipases” in public genomes from Agaricales has brought to light that the fungi with different habitats and life styles have different lipases arsenals. Analysis of the evolutionary history of these putative proteins revealed the increased length of the lid region and size of the substrate-binding pocket area across the evolution time, suggesting the specialization of these enzymes for certain substrates and their loss of promiscuity. Despite the consistency of the data showed by ancestral sequences reconstruction, it is often difficult to obtain a highly accurate sequence. Thus, the heterologous expression of some of these candidates would be interesting not only as a way to corroborate their real activities, but also to establish a platform for their artificial evolution in order to create enzymes endowed with novel capabilities.

## Additional files

Additional file 1: Number of putative versatile-lipases found in fungi from the order Agaricales, family and habitat of each fungus. (XLSX 12 kb)
Additional file 2: Putative versatile-lipases identified in public genomes from fungi of the order Agaricales. (XLSX 17 kb)
Additional file 3: Sequences alignment of the putative ancestral versatile lipases identified. (FAS 5 kb)
